# Pleiotropy drives evolutionary repair of the responsiveness of polarized cell growth to environmental cues

**DOI:** 10.3389/fmicb.2023.1076570

**Published:** 2023-07-14

**Authors:** Enzo Kingma, Eveline T. Diepeveen, Leila Iñigo de la Cruz, Liedewij Laan

**Affiliations:** Department of Bionanoscience, Kavli Institute of NanoScience, Faculty of Applied Sciences, Delft University of Technology, Delft, Netherlands

**Keywords:** laboratory evolution, adaptation, phenotypic plasticity, fluctuating environment, cell architecture, cell polarity

## Abstract

The ability of cells to translate different extracellular cues into different intracellular responses is vital for their survival in unpredictable environments. In *Saccharomyces cerevisiae*, cell polarity is modulated in response to environmental signals which allows cells to adopt varying morphologies in different external conditions. The responsiveness of cell polarity to extracellular cues depends on the integration of the molecular network that regulates polarity establishment with networks that signal environmental changes. The coupling of molecular networks often leads to pleiotropic interactions that can make it difficult to determine whether the ability to respond to external signals emerges as an evolutionary response to environmental challenges or as a result of pleiotropic interactions between traits. Here, we study how the propensity of the polarity network of *S. cerevisiae* to evolve toward a state that is responsive to extracellular cues depends on the complexity of the environment. We show that the deletion of two genes, *BEM3* and *NRP1*, disrupts the ability of the polarity network to respond to cues that signal the onset of the diauxic shift. By combining experimental evolution with whole-genome sequencing, we find that the restoration of the responsiveness to these cues correlates with mutations in genes involved in the sphingolipid synthesis pathway and that these mutations frequently settle in evolving populations irrespective of the complexity of the selective environment. We conclude that pleiotropic interactions make a significant contribution to the evolution of networks that are responsive to extracellular cues.

## Introduction

Polarity establishment, the ability to generate an asymmetric distribution of cellular constituents, plays an important role in many of the biological functions that are observed throughout the tree of life (Piroli et al., 2019). The dynamics of polarity establishment is regulated by an intricate network of molecular interactions, many of which are evolutionary conserved (Etienne-Manneville, 2004; Thompson, 2013; Chiou et al., 2017). What allows these networks to be versatile while maintaining a relatively high degree of conservation is their ability to generate different responses to various extracellular signals (Dickinson, 2008; Saito, 2010; Waltermann and Klipp, 2010). This feature makes it possible for the polarized appearance of cells to vary between environmental contexts (Granek et al., 2011).

Responsiveness to extracellular signals requires the integration of the polarity network with other molecular networks that either directly or indirectly translate these signals into an intracellular response (Saito, 2010; Waltermann and Klipp, 2010; Granek et al., 2011; Broach, 2012; Mutavchiev et al., 2016; Salat-Canela et al., 2021). An issue of integrated networks is that the decrease in modularity that arises when networks become coupled can frustrate evolvability (Fisher, 1930; Wagner and Altenberg, 1996; Kirschner and Gerhart, 1998; Hartwell et al., 1999; Wagner and Zhang, 2011). Because coupled networks become interdependent, the likelihood that a single mutation affects multiple phenotypic traits, an effect known as pleiotropy (Fisher, 1930; Wagner and Zhang, 2011), increases. Such pleiotropic effects are indeed frequently reported for genes involved in the establishment of cell polarity (Bauer et al., 1993; Zou et al., 2008; Prunskaite-Hyyryläinen et al., 2014). As antagonistic effects, where a mutation that is beneficial to one trait negatively affects a second trait (Paaby and Rockman, 2013; Austad and Hoffman, 2018; Mauro and Ghalambor, 2020), are considered to be more common than synergistic effects, pleiotropy is generally expected to constrain the number of accessible mutations during evolution in complex environments that select on multiple traits (Fisher, 1930; Waxman and Peck, 1998; Orr, 2000; Welch and Waxman, 2003). In turn, evolution in simple environments may not be constrained by pleiotropic interactions, but can instead lead to the deterioration of networks regulating unused traits (Rose and Charlesworth, 1980; MacLean et al., 2004; Qian et al., 2012; Fraebel et al., 2017). Thus, the molecular details of adaptive evolution of the polarity network are expected to depend on the environment: complex environments only allow mutations that preserve the integrity of coupled networks, while the released constraint in simple environments allows the system to explore alternative evolutionary pathways, but at the cost of the disintegration of unused networks and a loss of the ability to respond to environmental cues. However, whether these theoretical expectations form a general rule for the evolution of pleiotropically connected traits and if exceptions can be identified based on the molecular basis of their pleiotropic interactions is still a point of discussion (Agrawal and Stinchcombe, 2009; Jerison et al., 2020).

An attractive system to study the effect of pleiotropic interactions on the evolution of cell polarity is the yeast *Saccharomyces cerevisiae*. *S. cerevisiae* has adopted asymmetric cell division as its main mode of proliferation and must therefore establish an axis of polarity once per cell cycle (Martin and Arkowitz, 2014; Chiou et al., 2017). In addition, its polarity network is integrated with several different signaling networks to allow different growth modes in response to environmental cues, such as those that signal cell cycle progression (Yoshida and Pellman, 2008), filamentous growth (Cullen and Sprague, 2012) and the activation of stress response pathways (Saito, 2010; Waltermann and Klipp, 2010). Here, we study whether the polarity network can restore its coupling to signaling networks after this coupling has been lost due to a genetic perturbation and how this restoration depends on selective pressures from the environment. In addition, we discuss whether known connections between the polarity network and other signaling pathways are able to explain our observed patterns of adaptation. To do this, we use a *bem3*Δ*nrp1*Δ strain of *S. cerevisiae* that has been demonstrated to be defective in polarity establishment during vegetative growth. We show that this genetic perturbation also disrupts the responsiveness of the polarity network to an environmental shift that induces cells to change their metabolic program. Using a combination of experimental evolution and whole-genome sequencing, we find that adaptive mutations that restore the responsiveness of the polarity network to this environmental insult emerge frequently and reproducibly in evolving populations and that their occurrence is surprisingly insensitive to the complexity of the environment.

## Results

### Deletion of BEM3 and NRP1 distorts cellular adaptation during the diauxic shift

The combined deletion of *BEM3* and *NRP1* has been shown previously to cause defects in polarity establishment that exceed the summed effects of their individual deletion (Laan et al., 2015), meaning they exhibit epistasis (Phillips, 2008). The existence of epistatic interactions between these mutations suggests a functional relation between Bem3 and Nrp1. This is surprising, because while Bem3 is known as a GTP Activating Protein (GAP) for Cdc42, the master regulator of cell polarity (Etienne-Manneville, 2004), Nrp1 has never been implicated to be involved in polarity establishment before. Instead, based on the current knowledge about its function, Nrp1 is best described as a prion forming protein that localizes to stress granules formed under conditions of glucose stress (Buchan et al., 2008; Kroschwald et al., 2015). This led us to hypothesize that the deletion of *BEM3* and *NRP1* may have consequences for the ability of the polarity network to respond to environmental cues that signal different growth modes.

We tested this hypothesis in the context of the ability of *S. cerevisiae* to perform diauxic growth between glucose and ethanol. In the presence of extracellular glucose, *S. cerevisiae* maintains a rapid mode of growth by alcoholic fermentation of glucose. The ethanol produced during alcoholic fermentation can be used as an alternative energy source when extracellular glucose drops below a critical level, but only in the presence of extracellular oxygen. The transition from the fermentation of glucose to the respiration of ethanol, a growth phase known as the diauxic shift, is characterized by several physiological changes (Galdieri et al., 2010), which includes changes in the polarized distribution of the actin cytoskeleton (De Virgilio and Loewith, 2006; Galdieri et al., 2010).

**FIGURE 1:**
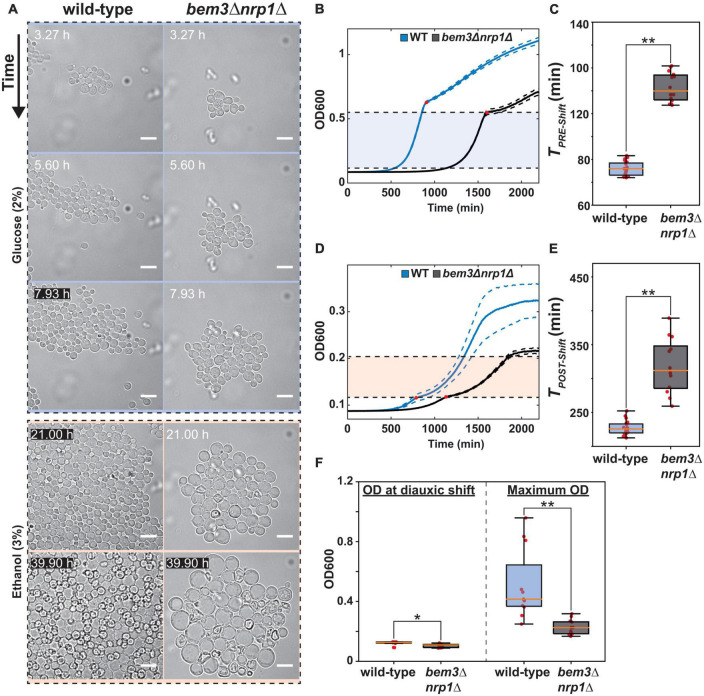
Deletion of *bem3*△*nrp1*△ causes defects in pre- and post-diauxic growth. **(A)** Time-lapse series of the diauxic shift. The WT and *bem3*△*nrp1*△ strain were subjected to a switch from 2% glucose media to 3% ethanol media after 8 h in 2% glucose media. The images show that while the WT strain is able to resume growth, the *bem3*△*nrp1*△ cells increase in size without producing daughter cells. Scale bars represent 10 μm **(B)** Growth curves of a WT (blue) and the *bem3*△*nrp1*△ mutant (black) when grown in 2% glucose media. This data was used to obtain a measure for *T*_*PRE–shift*_. Red dots indicate the point of diauxic shift, dashed lines represent the Standard Error of the Mean (SEM). **(C)** Doubling time of the WT strain and the *bem3*△*nrp1*△ mutant during growth before the diauxic shift is entered (*T*_*PRE–shift*_). **(D)** Growth curves of a WT (blue) and the *bem3*△*nrp1*△ mutant (black) when grown in 0.1% glucose media. This data was used to obtain a measure for *T*_*POST–shift*_. Red dots indicate the point of diauxic shift, dashed lines represent the SEM. **(E)** Doubling time of the WT strain and the *bem3*△*nrp1*△ mutant during growth after passing through diauxic shift is entered (*T*_*POST–shift*_). **(F)** Comparison of the OD at which the *bem3*△*nrp1*△ mutant and the WT strain enter the diauxic shift and their OD at stationary phase when grown in YP + 0.1% glucose. The plot shows that while both strains enter diauxic shift at around the same density, the final density of the populations differ. *p-value < 0.05, **p-value < 0.005, Welch *t*-test.

We qualitatively determined the effects of deleting *BEM3* and *NRP1* on the coupling of (diauxic growth) glucose sensing to cell polarity by imaging *bem3*Δ*nrp1*Δ cells during the diauxic shift (Figure 1A). The diauxic shift was induced by switching from growth media containing glucose as the sole carbon source to one where ethanol was the sole carbon source using a microfluidic device. A wild-type strain subjected to these conditions displayed the expected behavior, which consisted of rapid growth on glucose followed by a short growth pause at the time of the media switch, after which growth was resumed on ethanol media, but at a slower rate compared to growth on glucose media (Brauer et al., 2005). Overall, *bem3*Δ*nrp1*Δ cells followed the same pattern that we observed for the wild-type cells, but critically failed to produce buds during growth on ethanol media. Instead, isotropic growth was sustained in these cells up to the point where it induced cell death by lysis. Based on the link between polarity defects and an increase in cell size, we deduced that our observations for the *bem3*Δ*nrp1*Δ phenotype are the result of the inability of the polarity network to respond appropriately to the physiological changes that occur during the diauxic shift. Specifically, while *bem3*Δ*nrp1*Δ mutants are generally less fit than the wild-type strain, the cellular defect that leads to a lower fitness differs between conditions of standard vegetative growth and conditions where the cells must respond to the diauxic shift. During vegetative growth (2% glucose in Figure 1A) *bem3*Δ*nrp1*Δ cells proliferate, but do so at a slower rate than wild-type cells. After the media switch (transition from 2% glucose to 3% ethanol in Figure 1A) *bem3*Δ*nrp1*Δ cells enlarge, but are unable to divide.

Next, we quantified the effect of deleting *BEM3* and *NRP1* on the diauxic shift using Optical Density (OD) measurements of population growth in order to obtain growth curves for each strain (Figures 1B, D). The diauxic shift was clearly visible in the growth curves as a transition period between two exponential growth phases with different growth rates. For technical reasons (see Supplementary Figure 1), we used media containing a high glucose concentration (2%) to quantify growth before the diauxic shift and a lower glucose concentration (0.1%) to quantify growth after the diauxic shift. We extracted the growth rate during the exponential phase before and after the occurrence of the diauxic shift by calculating the slope of the linear portion of the growth curve when plotted on a semi-log scale. These values were subsequently converted into their corresponding doubling times (*T*_*PRE–shift*_ and *T*_*POST–Shift*_). This analysis revealed that *bem3*Δ*nrp1*Δ populations have a significantly longer doubling time than the wild-type both before and after onset of the diauxic shift (Figures 1C, E). While it is expected that the overall fitness defect of *bem3*Δ*nrp1*Δ mutants will lead to longer doubling times both before and after the diauxic shift, we argue based on our microfluidic experiment (Figure 1A) that the physiological cause that leads to a lower doubling time is different between the two conditions. Before the diauxic shift, *bem3*Δ*nrp1*Δ cells divide at a slower rate than the wild-type strain due to a defect in polarity establishment which causes a longer *T*_*PRE–shift*_. In contrast, *T*_*POST–Shift*_ is affected by both the slower division rate *and* the higher death rate of *bem3*Δ*nrp1*Δ cells as the polarity defect becomes much more severe at the onset of the diauxic shift. In support of this idea, we found that *bem3*Δ*nrp1*Δ populations stop growing at a significantly lower OD than wild-type populations after the diauxic shift (ratio wild-type: *bem3*Δ*nrp1*Δ = 2.25, Figure 1F), while both strains enter the diauxic shift at approximately the same density (ratio wild-type: *bem3*Δ*nrp1*Δ = 1.15, Figure 1F). We therefore interpret these results as indications that the defects in polarity establishment caused by the deletion of *BEM3* and *NRP1* makes the polarity network insensitive to environmental cues that signal the onset of the diauxic shift. The loss of responsiveness to these cues causes an inability to establish a polarity site when the physiological changes related to the diauxic shift have taken place, leading to prolonged isotropic growth and an increase in cell size.

### Recoupling of polarity establishment to sensing networks does not require a complex environment

**FIGURE 2:**
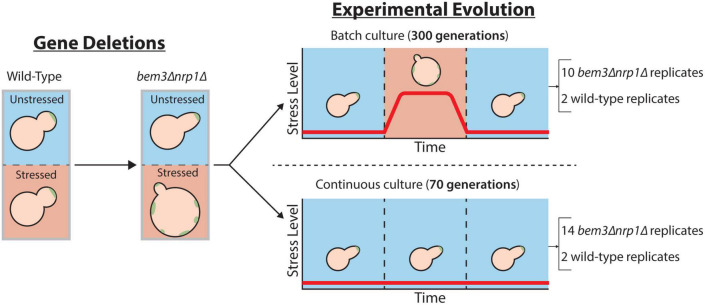
Overview of the scheme for experimental evolution. A polarity mutant that displays sensitivity to environmental stress is obtained after the deletion of the genes *BEM3* and *NRP1*. To assess the role the environment plays during the evolution of a network that is responsive to environmental signals, this mutant is evolved in an environment in which the stress level fluctuates batch culture and an environment where the stress level is constant continuous culture.

We sought to determine whether the environment is the decisive factor that controls the adaptive value of restoring the cellular response to the diauxic shift during evolution. To do this, we took an experimental approach and evolved several parallel wild-type and *bem3*Δ*nrp1*Δ populations in two frequently used set-ups for experimental evolution (Figure 2). In the first set-up, the *batch culture*, nutrient levels vary over time and cells experience periods of glucose depletion several times throughout the experiment (Brauer et al., 2005; Gresham and Dunham, 2014). Mutations that allow cells to correctly coordinate the physiological changes necessary to pass through the diauxic shift with those that regulate polarity establishment are therefore expected to be beneficial during evolution in a batch culture set-up, as this extends the overall number of progeny that a cell can produce before each passage. In the second set-up, the *glucose limited continuous culture*, nutrient concentrations remain constant after a steady state is reached and growth is maintained at a constant rate (Brauer et al., 2005; Gresham and Dunham, 2014). These constant environmental conditions have the consequence that cells do not induce the majority of the cellular responses that are associated with the diauxic shift (Brauer et al., 2005). Thus, the ability to perform diauxic growth appears as a dispensable trait during evolution in a continuous culture. Based on the theoretical assumptions that traits that do not experience selective pressure (1) tend to deteriorate and (2) are unlikely to fix mutations that improve their function during evolution, we expect restoration of diauxic growth by *bem3Δnrp1* populations to emerge only during batch culture evolution.

We evolved a total of 14 *bem3*△*nrp1*△ populations and 2 wild-type populations in the glucose limited continuous culture for 70 generations. The parameter *T*_*PRE–Shift*_ was used as a proxy for adaptations that restore the polarity defect caused by the deletion of *BEM3* and *NRP1*, but that do not necessarily improve the ability of the polarity network to respond to cues that signal the onset of the diauxic shift. Alternatively, *T*_*POST–Shift*_ was used as a proxy for adaptations that improve the response of cells to the diauxic shift. The values of *T*_*PRE–Shift*_ and *T*_*POST–Shift*_ of the evolved cell lines were determined by reviving the evolved population from a frozen stock and measuring the change in OD over time in media containing 2% and 0.1% glucose, respectively. This procedure is the same as what was done to determine *T*_*PRE–Shift*_ and *T*_*POST–Shift*_ for the ancestral wild-type and *bem3*△*nrp1*△ populations (see Figures 1B, D, and the section “Materials and methods”).

**FIGURE 3:**
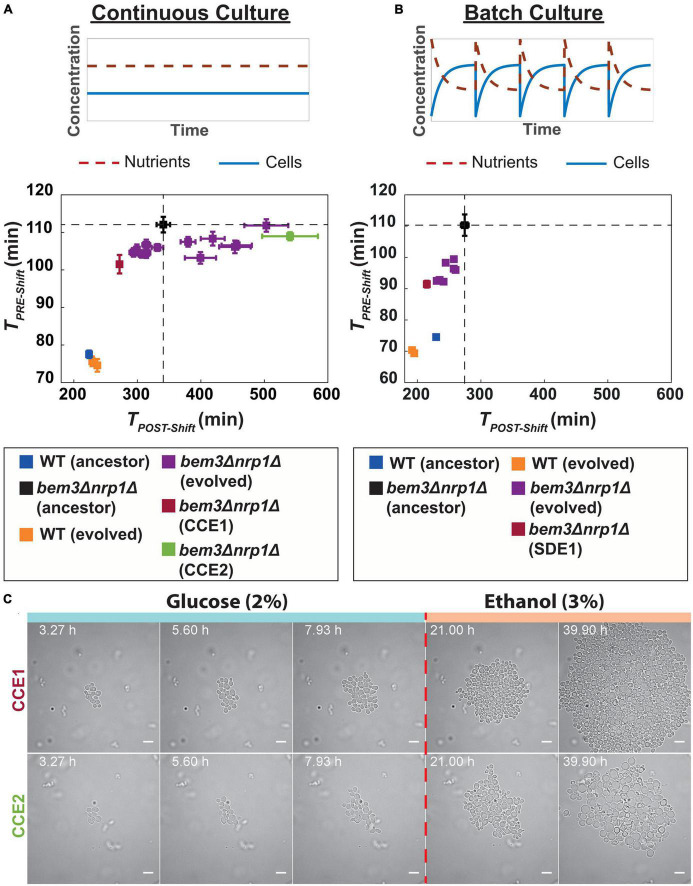
Experimental evolution of *bem3*△*nrp1*△ mutants in a constant and variable environment. **(A)** (Top) In a continuous culture, both nutrient concentration and cell density remain constant over time. (Bottom) Scatter plot of *T*_*PRE–Shift*_ against *T*_*POST–Shift*_ for 14 evolved *bem3*△*nrp1*△ lines and 2 wild-type populations after 70 generations of evolution in a continuous culture. Dashed lines indicate the values of *T*_*PRE–Shift*_ and *T*_*POST–Shift*_ of ancestral *bem3*△*nrp1*△ strain. Error bars show the SEM. **(B)** (Top) In a batch culture there are periodic fluctuations over time in nutrient concentration and cell density. (Bottom) Scatter plot of *T*_*PRE–Shift*_ against *T*_*POST–Shift*_ for 8 evolved *bem3*△*nrp1*△ lines and 2 WT lines after 300 generations of evolution in a batch culture. Dashed lines indicate the values of *T*_*PRE–Shift*_ and *T*_*POST–Shift*_ of ancestral *bem3*△*nrp1*△ strain. Error bars show the SEM. **(C)** Time-lapse of evolved lines CCE1 and CCE2 (continuous culture) during a sudden switch from 2% glucose media to 3% ethanol media (dashed red line). The images show that evolved line CCE1 contains cells that have a response to this environmental change that is phenotypically similar to the response of the WT strain. Evolved line CCE2 has a response that resembles the response of the ancestral *bem3*△*nrp1*△, but with a smaller increase in cell size (see Figure 1A). Scale bars represent 10 μm.

Comparison of *T*_*PRE–Shift*_ between the evolved populations and their ancestor (Figure 3A) revealed that all evolved populations had either a similar or lower value for *T*_*PRE–Shift*_ relative to their ancestor. In contrast, we find that for *T*_*POST–Shift*_ half of the evolved *bem3*△*nrp1*△ populations (7/14) had a lower doubling time, while the other half (7/14) had a longer doubling time relative to their ancestor, indicating that changes in diauxic growth do not affect fitness in a continuous culture. A similar trend for *T*_*PRE–Shift*_ and *T*_*POST–Shift*_ was visible for our two evolved wild-type populations. Taken together, these observations support our initial view that a continuous culture selects for faster vegetative growth, but not diauxic growth.

The finding that some of *bem3*△*nrp1*△ populations evolved in the continuous culture show improvements in *T*_*POST–Shift*_ could be explained by a possible interdependence of *T*_*PRE–Shift*_ and *T*_*POST–Shift*_: improvements in *T*_*PRE–Shift*_ may be caused by mutations that increase the overall rate of cell division and these mutations will therefore also lead to improvements in *T*_*POST–Shift*_. However, these mutations do not necessarily also resolve the high death rate of *bem3*△*nrp1*△ mutants at the start of the diauxic shift (Figure 1A), which may be a major factor that determines *T*_*POST–Shift*_. To verify that a decrease in *T*_*POST–Shift*_ relates to adaptations that resolve the high death rate, we imaged cells from the evolved population with the lowest (fastest growing, CCE1) and highest (slowest growing, CCE2) value for *T*_*POST–Shift*_ during the diauxic shift (Figure 3C) using the same microfluidic set-up we used in Figure 1. In agreement with our expectations, the results showed that the phenotype of CCE1 after switching to ethanol media was qualitatively more similar to that of our ancestral wild-type strain and CCE1 cells were able to resume proliferation after the diauxic shift. Alternatively, the phenotype of CCE2 was more similar to the ancestral *bem3*△*nrp1*△ strain, as CCE2 cells enlarged and were frequently unable to divide after the onset of the diauxic shift.

We evolved 8 *bem3*△*nrp1*△ and 2 wild-type populations in a batch culture with a daily passaging procedure. We initially maintained the same number of generations for evolution as we had done for the continuous culture (70 generations), but after assessing our proxies for fitness we were unable to identify any significant changes in the values of *T*_*PRE–Shift*_ and *T*_*POST–Shift*_ between the evolved populations and their ancestors (see Supplementary Figure 2). We assumed that this is due to the frequent population bottlenecks that occur during the passaging of the populations, which can slow down the rate of adaptation by purging beneficial mutations from the population (Wein and Dagan, 2019). We provide an estimate of the effect of population bottlenecks on the fixation dynamics of beneficial mutations in Supplementary Section 1, which shows that bottlenecks vastly increase the expected number of generations that are required before a beneficial mutation that fixates in the population will emerge. To compensate for this effect of population bottlenecks, we allowed our batch culture experiment to run for an additional 230 generations such that the total number of generations was 300.

We found that all evolved populations grew faster than their ancestors, both before and after the diauxic shift (Figure 3B). The fact that we do not observe populations that evolve toward a state where the doubling time after the diauxic shift becomes longer suggests that these pathways are inaccessible during evolution in a batch culture. Taken together, these results imply that the environmental variability that exists in the batch culture imposes constraints on the diauxic growth pattern that can be attained during evolution, allowing only those where growth on both nutrients is improved, while the stable environment of the continuous culture releases some of these constrains. As a result, phenotypes that have evolved to perform well during the diauxic shift, presumably through evolutionary repair of the polarity defects caused by deleting *BEM3* and *NRP1*, only reproducibly emerge in a batch culture. However, although the degree of reproducibility is lower, similar phenotypes do frequently evolve in a continuous culture. This indicates that evolutionary constraints imposed by the environment are not sufficient to explain the restoration of the responsiveness of the polarity network to cues of the diauxic shift during evolution.

### Populations with a restored responsiveness to extracellular cues accumulate mutations in genes related to the sphingolipid synthesis pathway

To understand the molecular basis of the different adaptations of *T*_*PRE–Shift*_ and *T*_*POST–Shift*_ we observed in our continuous and batch cultures, we performed Whole Genome Sequencing (WGS) on the 22 evolved *bem3*△*nrp1*△ lines and the 4 evolved wild-type controls and compared them to the genome of their wild-type ancestor (see section “Materials and methods”). We looked for patterns of parallel evolution by restricting our analysis to genes that were mutated in at least 2 different populations evolved in the same environment. This resulted in a total of 88 genes that acquired non-synonymous mutations or indels in 2 or more evolved populations (including the wild-type lines).

The most notable environment-specific mutations were the early stop codons in *WHI2* that frequently occurred in the populations evolved in the continuous culture: 12 out of 14 evolved *bem3*△*nrp1*△ and both evolved WT controls had mutated *WHI2*. Disruptive mutations in *WHI2* have also been reported in other experimental evolution studies that used nutrient limited continuous cultures (Kvitek and Sherlock, 2013; Hong and Gresham, 2014) and these mutations therefore likely provide a general advantage during adaptation to nutrient-limiting conditions.

**FIGURE 4:**
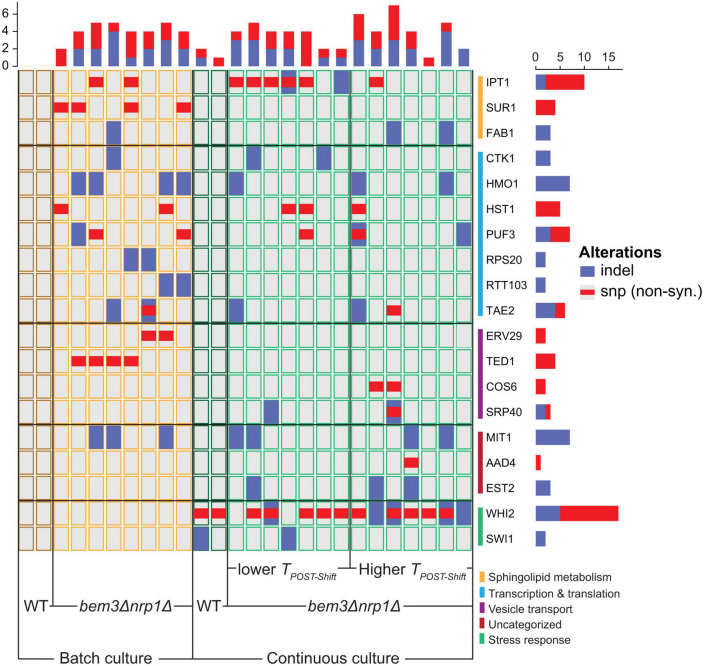
The mutational spectrum of different phenotypic subgroups that emerged after experimental evolution of *bem3△nrp1△* populations. The mutant-specific mutations found in each gene for evolved continuous culture lines that decreased their respiration rate, evolved continuous culture lines that increased their respiration rate and evolved batch culture lines. Genes are grouped according to their cellular process GO-term. All genes shown were only mutated in the *bem3△nrp1* populations and not in the wild-type populations, with the exception of *WHI2* and *SWI1*, which were also found to be mutated in the wild-type populations evolved in the continuous culture.

Because we saw the same phenotype emerge in the batch culture and continuous culture (populations that decreased *T*_*POST–Shift*_), we questioned whether the molecular basis of these adaptations were similar. In total, 22 genes were mutated in at least 2 of the *bem3*△*nrp1*△ lines evolved in each environment. We grouped these genes according to their Biological Process Gene Ontology (GO) annotation on the Saccharomyces Genome Database. This revealed that populations evolved in a continuous culture had more mutations in genes involved in the stress response, while populations from the batch culture had slightly more mutations in genes related to transcription and translation. We then split the evolved populations into two groups: those that evolved to decrease *T*_*POST–Shift*_ (7/14 populations of the continuous culture and 8/8 populations of the batch culture) and those that evolved to increase *T*_*POST–Shift*_ (7/14 populations of the continuous culture and 0/8 populations of the batch culture). Interestingly, populations with a decreased *T*_*POST–Shift*_ had more mutations in lipid metabolic genes than those that did not decrease *T*_*POST–Shift*_. Of the 14 *bem3*△*nrp1*△ populations that were evolved in the continuous culture, 6/7 populations with a decreased *T*_*POST–Shift*_ had mutations in the *IPT1*, while we only found mutations in this gene in 1/7 populations with an increased *T*_*POST–Shift*_ (Figure 4). In the batch culture populations, 2/8 had acquired mutations in *IPT1*, while 3/8 had mutations in *SUR1*. Notably, Ipt1 acts directly downstream of Sur1 in the pathway for the synthesis of complex sphingolipids (Thevissen et al., 2000; Dickson et al., 2006; Morimoto and Tani, 2015).

Based on this correlation we hypothesize that, after the deletion of Bem3 and Nrp1, the robustness of the polarity module during the diauxic shift can be (partially) restored by changes in the lipid composition of the plasma membrane. Interestingly, this strategy appears to be dominant for repairing the defect caused by the deletion of *BEM3* and *NRP1* regardless of whether diauxic growth is part of the selective environment.

## Discussion

The ability to respond to environmental cues is a crucial factor for the survival of organisms in complex environments. For example, studies have indicated that pathogens increase the likelihood of successfully infecting a host by adjusting their physiology to match the host’s circadian rhythm (Kahl Lisa et al., 2022). Here, we used a genetically perturbed strain of *S. cerevisiae* to investigate the contribution of the environment in shaping a polarity network that can translate the extracellular signals for diauxic growth into an intracellular response. We show that the deletion of *BEM3* and *NRP1* has previously unknown consequences for polarity establishment that diminishes its capacity to respond to these extracellular signals and impedes the ability of cells to successfully navigate through the diauxic shift. Which molecular mechanisms are affected by the deletion of *BEM3* and *NRP1* in such a manner that it leads to the observed phenotype are not addressed in this study. However, the results from several other studies that have looked at the relationship between environmental stress and cell morphology allow us to formulate a hypothesis on how the deletion of *BEM3* and *NRP1* causes the decoupling of cell polarity from diauxic growth. The link between cell morphology and environmental stress is frequently proposed to be a consequence of the loss of polarity of the actin cytoskeleton induced by stress factors (Sivadon et al., 1995; Balguerie et al., 2002; Uesono et al., 2004; Homoto and Izawa, 2018). Failure to repolarize the actin cytoskeleton following environmental stress, either due to the severity of the stress conditions (Homoto and Izawa, 2018) or due to the loss of a genetic component required for repolarization (Sivadon et al., 1995; Balguerie et al., 2002), results in enlarged cells. The similarity of these hypertrophied cells under conditions of environmental stress to the phenotype of *bem3*△*nrp1*△ mutants we observe during a transition from glucose-containing media to ethanol-containing media suggests they are caused by a defect in a similar pathway. Indeed, the depletion of glucose, one of the hallmark cues for entry into the diauxic shift (Brauer et al., 2005), has also been shown to cause the rapid and transient depolarization of actin in wild-type cells (Uesono et al., 2004; Vasicova et al., 2016). The repolarization of actin in the context of glucose depletion depends on the activation of the respiratory metabolism (Uesono et al., 2004), as cells with dysfunctional mitochondria do not repolarize actin (Uesono et al., 2004; Vasicova et al., 2016). Thus, one possibility is that the deletion of *BEM3* and *NRP1* causes defects in respiration. However, we consider this unlikely based on our observation that *bem3*△*nrp1*△ cells are still able to grow, although only isotropically, in media containing ethanol as the only carbon source. In addition, if mitochondrial dysfunction were the cause of the observed phenotype, mutations related to mitochondrial function would be expected to arise during our evolution experiments, but this was not the case.

Instead, our results suggest that the defects in diauxic growth of *bem3*△*nrp1*△ mutants are suppressed by mutations in the sphingolipid synthesis pathway. Interestingly, the genes (*IPT1* and *SUR1*) that were frequently mutated in evolved *bem3*△*nrp1*△ populations with a (partially) restored ability to pass through the diauxic shift are also known to suppress the sensitivities to stress and starvation that arise after the deletion of genes that encode for the amphiphysin-like proteins Rvs161 and Rvs167 (Desfarges et al., 1993; Balguerie et al., 2002). Rvs161 and Rvs167 have a direct role in regulating the polarity of the actin cytoskeleton (Amberg et al., 1995; Munn et al., 1995; Sivadon et al., 1995, 1997; Breton and Aigle, 1998) and their loss causes defects in the depolarization and repolarization dynamics of actin during stress in an equivalent manner as has been described for glucose stress in the section above (Crouzet et al., 1991; Bauer et al., 1993; Sivadon et al., 1995). Suppression of these defects through the deletion of *IPT1* or *SUR1* has been reported to act by preventing the full depolymerization of actin under stressful conditions (Balguerie et al., 2002), thereby relieving some of the consequences of an inability to repolarize actin. Extrapolating these findings to *bem3*△*nrp1*△ mutants, this implies that the evolutionary repair of diauxic growth in *bem3*△*nrp1*△ populations acts by directly modulating actin dynamics using sphingolipid synthesis as a control knob. Similarly, the pleiotropic effects resulting from the deletion *BEM3* and *NRP1* are therefore likely a consequence of the dual role of actin in polarized growth and stress response pathways (Ho and Bretscher, 2001; Leadsham et al., 2010; Smethurst et al., 2014) that couples polarity establishment to the diauxic shift.

It remains unclear why the consequences of deleting *BEM3* and *NRP1* are different for the actin dynamics required during vegetative growth and the actin dynamics under stressed conditions. Much alike to what happens under conditions of stress, the actin cytoskeleton must depolarize and repolarize during the cell cycle to switch between modes of isotropic and polarized growth (Lew and Reed, 1993; Welch et al., 1994; Pruyne and Bretscher, 2000; Ahn et al., 2001; Bi and Park, 2012). However, our results show that polarized growth during the vegetative cell cycle is not strongly affected by the deletion of *BEM3* and *NRP1*, while polarized growth after the stress response of the diauxic shift is strongly diminished. This suggests that cell cycle-related polarization of actin may be regulated by a different pathway than the polarization of actin during the stress response. We find that, despite that they may be regulated by different pathways, the ability to perform polarized growth in both contexts can be restored by mutations in genes related to sphingolipid synthesis. Surprisingly, the fixation of these mutations that restore both vegetative and diauxic growth does not strongly depend on the complexity of the environment. Instead, we frequently see them emerge in populations evolved under constant conditions where improved diauxic growth appears to have no selective benefit, as is supported by our result that nearly all populations evolved in the continuous culture inactivate *WHI2*, which encodes for a protein that initiates the stress response during nutrient depletion (Sudbery et al., 1980; Saul et al., 1985; Radcliffe et al., 1997; Kaida et al., 2002). Interestingly, a recent study investigating the adaptive response of *Escherichia coli* to different temperature fluctuation regimes also found that the same mutations frequently evolve in parallel in a manner that does not depend on the dynamics of the selective environment (Lambros et al., 2021). A large-scale phenotypic assay revealed that the evolved strains generally became closer to the phenotype of their ancestor under a large number of conditions, leading to the hypothesis that an innate evolutionary response of an organism in a stressful environment is to evolve in such a way that their physiology resembles that of their (fitter) ancestor in unstressed conditions. Overall our results agree with this hypothesis, as we find that genetically perturbed cells frequently evolve to better match the cellular response of their ancestor, even in unseen environmental conditions. Possible explanations for why the fixation of mutations that restore the cellular response to conditions beyond those experienced during adaptation would be preferred are that (1) these mutations might occur more frequently in the population because they constitute mutational hotspots or (2) their fixation is purely driven by the fitness benefit that they confer to vegetative growth and the restored diauxic growth is merely a side effect of a pleiotropic interaction network. In conclusion, our results demonstrate that the evolution of interaction networks that can sense and respond to different environmental signals should not always be interpreted as adaptive, but may instead be a consequence of a strong integration between different interaction networks regulating different cellular functions. Such an integration of different interaction networks may also be able to explain observations of the seemingly purposeless emergence of phenotypic plasticity, the ability of an organisms to adjust its phenotype to its environment, during evolution in constant environmental conditions (Fraebel et al., 2020).

## Materials and methods

### Yeast strains and media preparation

All strains used in this study are derived from the W303 background and are *MATa* haploid cells. We used yLL132a as our WT strain and yLL143a as our *bem3*△*nrp1*△ strain (Laan et al., 2015), which has the same genetic background as yLL132a, but with *BEM3* and *NRP1* replaced with, respectively, the natMX4 (clonNAT-Nourseothricin resistance) and hphMX4 (Hygromycin B resistance) cassettes. For batch culture evolution experiments, standard rich media (10 g/L Yeast Extract, 20 g/L Peptone and 20 g/L Dextrose) was used and was prepared by dissolving 50 g/L from a premixed batch of ingredients (Sigma-Aldrich) in H_2_O. For chemostat evolution experiments the same premix was used, but supplemented with 19 g/L extra Yeast Extract and 9.5 g/L extra Peptone to obtain a final dextrose concentration of 1 g/L. A total of 0.1 mg/mL Ampicillin was added to the chemostat media as a safeguard against bacterial contamination. Microscopy experiments were performed in Synthetic Complete (SC) media prepared from Complete Supplement Mixture without amino acids, riboflavin and folic acid (750 mg/L), Yeast Nitrogen Base (6.9 g\L) and either Dextrose (2%w/v) or Ethanol (3%v/v) as a carbon source. All media was filter sterilized to avoid degradation of components during autoclaving.

### Experimental evolution of continuous cultures

#### Multiplexed chemostat array set-up

We performed our evolution experiments in a dextrose limited chemostat environment by setting up a multiplexed chemostat array of 16 cultures according to the protocol from Miller et al. (2013). YP 0.1%D media was filter sterilized directly into a 10 L glass carboy. During the run, fresh media was provided to the cultures from this carboy by using a peristaltic pump fitted with Marprene tubing. The correlation between rotation speed and media flow rate was empirically determined by measuring the effluent volume at different rotation speeds. Aquarium pumps were used to maintain the positive pressure inside the culture chambers required for the removal of excess culture volume, to keep the cultures aerated and mixed. To minimize evaporation and maintain sterility, air from the pumps was first routed through gas washing bottles and 0.45 μm PFTE filters before entering the culture chambers. The temperature was regulated at 30°C using heat bocks.

#### Initialization of multiplexed arrays

We initialized our multiplexed chemostat arrays by allowing the culture chambers to fill with media until the volume exceeded 20 mL. We dissolved cells from a glycerol stock in YP 0.1 %D media and used to inoculate the cultures by aseptically injecting 4 mL into each culture chamber. In total, 14 *bem3*△*nrp1*△ cultures and 2 WT cultures were inoculated using this procedure. With the peristaltic pump turned off and the aquarium pumps turned on, the *bem3*△*nrp1*△ cultures were left to grow for 4 days and the WT cultures were left to grow for 2 days until they reached saturation (batch phase growth). After the cultures reached saturation, the culture volume was set at 20 ± 1 mL while performing the zero time point sampling.

#### Sampling regimen

All cultures were sampled twice a week. Samples were taken by replacing the effluent bottles with sterile sampling bottles and collecting the effluent on ice over a period of approximately 2 h. Directly after sampling, 1 mL of each collected sample was mixed with 500 μL glycerol and stored at −80°C. Optical Density (OD) measurements at 600 nm were taken of each sample in 10 mm plastic cuvettes using a photospectrometer (Nanorop 2000C). When necessary, samples were diluted with YP to obtain a final OD of between 0.1 and 1.5. All samples were diluted in the same media used for blanking the photospectrometer. Effluent volumes were measured daily with a graduated cylinder from which the volume could be read with 0.5 ml precision. On days that sampling took place, the effluent volume of samples was determined after the standard procedure for sampling (glycerol stocks and OD measurements).

#### Calculation of dilution rates and generation times

We calculated the dilution rate *D* of each sample in our multiplexed chemostat array from the effluent volume using the following formula:


$$D=\frac{V_{E\,f\,f}}{t\cdot V_{C\,u\,l\,t}}.$$

Here, *V*_*Eff*_ is the measured effluent volume, *t* is the time that has passed since the last sampling and *V*_*Cult*_ is the culture volume. At steady state, the growth rate equals the dilution rate [63], allowing the number of generations *G* that have passed to be calculated by:


$$G=t\cdot \frac{D}{\ln2}.$$

### Experimental evolution of batch cultures

Batch culture evolution experiments were started with 10 *bem3*△*nrp1*△ and 2 WT cultures. The cultures were derived from a single *bem3*△*nrp1*△ and a single WT liquid culture initiated from a glycerol stock and grown to saturation for 2 days in YP 2 %D in a roller drum (set at 40 RPM) at 30°C. After the cultures reached saturation, 10 μL of each starter culture was diluted into 10 mL of fresh YP 2 %D and were placed back into the roller drum. The cultures were diluted by 10 μL into 10 mL of fresh YP 2 %D every 24 ± 2 h. After each dilution, the OD at 600 nm of the remaining culture was measured using the same procedure as described above for the chemostat evolution experiment. Because batch cultures involve frequent population bottlenecks that can reduce genetic variation and possibly purge beneficial mutations (Gresham and Dunham, 2014; Gresham and Hong, 2014), it might take longer for an adaptive mutation to settle in the population. To compensate for this effect, the number of generations that the populations were evolved in a batch culture setting was increased to 300 generations (an additional 230 generations compared to the populations evolved in a continuous culture).

### Growth curve measurements

Growth curves were obtained by measurements using a plate reader (Tecan Infinite 200 Pro). Cells were inoculated from a glycerol stock in YP 0.1 %D liquid media and grown to saturation for 2 days in a roller drum at 30°C. On the day of the measurement, the saturated cultures were diluted 1000X into either fresh YP 0.1 %D or fresh YP 2 %D, depending on whether we wanted to measure the doubling time before (*T*_*PRE–Shift*_) or after (*T*_*POST–Shift*_) the diauxic shift. A total of 100 μL of this culture was pipetted into each well of a sterile 96-well plate (Nunc™Edge 2.0, Thermo Scientific™) with the edge moats filled with 1.7 mL of sterile H_2_O. Each plate contained multiple technical replicates of each sample. As a control for contamination and to allow for background subtraction for downstream processing, 8 wells were filled with blank medium. Measurements were taken during incubation at 30°C in the plate reader using the following protocol: First, the cells were shaken for 1000 s (linear shaking, 1 mm amplitude) without measurement. After this, the absorbance of each well was measured every 7 min with intermittent shaking (260 s, linear, 1 mm amplitude) for 48 h.

### Growth parameter calculations

Doubling times for pre-diauxic (*T*_*PRE–Shift*_) and an post-diauxic (*T*_*POST–Shift*_) growth were extracted from the growth curve measurement in YP 2 %D and YP 0.1 %D, respectively. First, the measured OD values were blanked using the time average value of one of the wells containing blank media. Then, the data was converted to semi-log data by taking the natural logarithm of the blanked OD values. A home written MATLAB script was used to fit a line to the linear portion of the semi-log data to obtain the growth rate during pre-diauxic or post-diauxic growth. These growth rates were converted into doubling times using the following relation:


$$T_{d}=\frac{\ln2}{\mu },$$

where *T_d_* is the doubling time corresponding to the growth rate μ obtained from the slope of the linear fit.

### Microscopy and microfluidics

Cells were grown to log phase in SC media containing 2% dextrose. Clumps of cells were dissociated prior to imaging by sonicating (Q500 Sonicator, QSonica) in a sealed Eppendorf tube using a cup horn at 70% amplitude for 2 min (cycle of 30 s pulse on, 15 s pulse off). After sonication, each sample was diluted to the same optical density in fresh Synthetic Complete media containing 2% dextrose. Cells were trapped in a microfluidic culture chamber (CellASIC ONIX Y04C-02, Merck–Millipore) after flushing the culture chambers with fresh media for 20 min using a pressure of 8 psi. Brightfield images were taken with a Nikon Eclipse Ti-E inverted Microscope using a 60x objective (Plan Apo λ 60X oil, NA: 1.40) with 1 min intervals. During imaging, cells were maintained in a constant flow of media using a pressure of 1 psi. Cells were subjected to a media switch by changing from an inlet with SC media containing 2% dextrose to an inlet with SC media with 3% ethanol after 8 h of imaging.

### DNA extraction, Illumina library preparation, and whole genome sequencing

We extracted genomic DNA from liquid cultures grown for two overnights for each of the 16 chemostat samples, 10 serial dilution samples and a non-evolved yLL132a ancestor with the MasterPure Yeast DNA Purification Kit (Epicentre, Madison, WI, USA) following the manufacturer’s protocol. We included a RNase A (Qiagen, Hilden, Germany) treatment step in the protocol and collected DNA in a final volume up to 30 μL H_2_O. We pooled up to three extractions per sample using the Genomic DNA clean and Concentrator kit (Zymo Research, Irvine, CA, USA), following the supplied protocol. We eluted DNA in a final volume of 30 μL. We assessed DNA quality by 0.8 % agarose gel electrophoresis and quantity by fluorometry using a Qubit 4.0 Fluorometer (Invitrogen, Carlsbad, CA, USA). Samples were individually barcoded and pooled into a single library with the NEB Next Ultra DNA Library Prep Kit (New England Biolabs, Ipswich, MA, USA) and sequenced on a HiSeq machine (Illumina, San Diego, CA, USA) by Novogene (Beijing, China).

### WGS data analysis

We first checked raw paired-end reads (150 bp) for quality with the FASTQC toolkit (version 0.11.7).^1^ We removed low quality ends (Quality scores <20; and first 9 bases of all reads), and removed duplicates with the FastX toolkit (version 0.0.14).^2^ We downloaded the R64-1-1 *S. cerervisiae* genome from the Saccharomyces Genome Database (SGD)^3^ and used it as our reference. We indexed the reference genome with the Burrows-Wheeler Aligner [BWA; version 0.7.17; (Li and Durbin, 2010)], and SAMtools [version 1.8; (Li and Durbin, 2009; Li, 2011)], and generated a dictionary with Picard (version 2.18.5).^4^ We mapped sequences from all samples individually to the reference with BWA-MEM sorted and indexed mapped reads into a BAM file with SAMtools. We performed multisample SNP calling and additional indexing with SAMtools and BCFtools (version 1.8). We plotted and checked statistics, e.g., TS/TV and quality of sites and read depth, with BCFtools. These statistics were used to filter out SNPs and Indels with low quality sites (QUAL > 30), low read depth (DP > 20), and variants in close proximity to gaps (SnpGAP 10). We annotated the VCF file with snpEff [version 4.3T; (Cingolani et al., 2012)] with R64- 1-1.86. We then retrieved variants (SNPs and indels) of interest through comparison of variants between the reference strain, the ancestor strains, and the evolved strains. We excluded variants that were different between R64 and all our W303 samples, as these merely display differences between the two genetic backgrounds [see e.g., (Ralser et al., 2012)]. Synonymous variants, variants in non-coding regions, and stop retained variants were excluded. Mutations in telomeric regions and in Long Terminal Repeats (LTRs) were excluded from analysis due to the natural variation that occurs in the genomic sequence of these regions. To find causative mutations, we looked for genes that mutated in at least two evolved lines, excluding those that appeared only in the mutant line(s) from one environment and the wild-type line(s) of the other environment. From the resulting list of genes, genes corresponding to dubious or uncharacterized Open Reading Frames (ORFs) were removed according to their description on SGD. Two genes (*RPS29B* and *ECM33*) had acquired the same mutation across all 22 parallel evolved *bem3*△*nrp1*△ lines that sweeped the population, suggesting that these mutations were acquired in the ancestor before the different cell lines were split. Although these mutations might have some fitness benefit in the *bem3*△*nrp1*△ background, they do not explain the adaptation we observe during our evolution experiments and we therefore excluded them from further analysis. We used the OncoPrint function from the ComplexHeatmap package (Gu et al., 2016) available in R (version 4.2.3) to visualize the relevant mutations in our evolved lines as a heatmap.

## Data availability statement

The data presented in this study are available under the CC0 license in the 4TU.ResearchData repository: https://doi.org/10.4121/01c1cf07-870a-4bc5-8b04-7df0919e0304.v1.

## Author contributions

LL and EK designed the research and wrote the manuscript. EK, ED, and LI executed the research. All authors contributed to the article and approved the submitted version.

## Footnotes

1
https://www.bioinformatics.babraham.ac.uk/projects/fastqc/

2
http://hannonlab.cshl.edu/fastx_toolkit/

3 www.yeastgenome.org (accessed September, 2018)

4
https://broadinstitute.github.io/picard/
