# Supplementary material for: Pleiotropy drives evolutionary repair of the responsiveness of polarized cell growth to environmental cues
Source: Front Microbiol. 2023 Jul 14;14:1076570. doi: 10.3389/fmicb.2023.1076570 (PMC10382278; doi:10.3389/fmicb.2023.1076570)
Supplement: Supplementary file 3 [file Data_Sheet_1.docx]

## **Supplementary section 1: Estimation of relative fixation times**

Serial dilution approaches for experimental evolution often include severe population bottlenecks which affect the fixation time of beneficial mutations. Here, we provide an estimate of how the fixation time of a beneficial mutation in a batch culture population relates to the fixation time of the same mutation in a continuous culture. The total fixation time $T_{fix}$ can be subdivided in the following two components:

$$T_{fix}=T_{m}+T_{s} , (1)$$

where $T_{m}$ is the time it takes for a mutation to emerge that will eventually fix and $T_{s}$ is the time it takes for this mutation to sweep the population. For the classical case of a large population of fixed size, it has been derived that the fixation probability of a beneficial mutation is proportional to the selective coefficient $s$ (Haldane, 1927). Because population size is large ($N_{c}\sim{10}^{9}$) and remains roughly constant in a continuous culture, we use this result to approximate the fixation probability of mutations for samples evolved in the chemostat:

$$P\left( chemostat \right)\approx2s. (2)$$

Which is valid for small values of $s$ ($s\ll1$). In the batch culture evolved samples, population size does not remain constant, but rather fluctuates during each serial passaging between the values $N_{0}$ ($\sim{10}^{6}$) and $N_{f}$ (${\sim10}^{9}$). Here, $N_{f}$ is the number of individuals just before reseeding the population in fresh media and $N_{0}$ is the number of individuals just after reseeding, such that $N_{0}=D\cdot N_{f}$ with $D$ the dilution factor. Wahl et al. (2002) have shown that periodic bottlenecks caused by serial passaging reduce the fixation probability by a factor ${D\left[ \ln D \right]}^{2}$, such that the fixation probability in a batch culture can be approximated as:

$$P\left( batch \right)\approx2s\cdot D\left[ \ln D \right]^{2}. (3)$$

If we now calculate the relative fixation probabilities between the two environment using the $D=0.001$ dilution factor we have used in our experiments, we find:

$$\frac{P(chemostat)}{P(batch)}\approx\frac{2s}{2s\cdot D\left[ \ln D \right]^{2}}=\frac{1}{D\left[ \ln D \right]^{2}}\approx21.$$

Thus, for an equal rate of beneficial mutations in both conditions we can expect that it will take approximately 21 fold more generations for a mutation to emerge that eventually fixes in the batch culture relative to the continuous culture. We can therefore write that

$$T_{m,batch}\approx21{\cdot T}_{m,chemostat}.$$

For the sweeping times (in generations) of mutations that fix in the batch culture, we use the approximation for large populations that are evolved through serial passaging given by Campos and Wahl (2009):

$$T_{s,batch}\approx\frac{2}{s}\ln N_{0}.$$

In the continuous culture the change in mutant frequency can be modelled as a Moran process of a well-mixed population, for which the fixation time is of the order (Antal and Scheuring, 2006; M Altrock and Traulsen, 2009; Tkadlec et al., 2019):

$$T_{s, chemostat}\approx\left( 1+\frac{2}{s} \right)\ln N_{c}\approx\frac{2}{s}\ln N_{c}, (4)$$

where we have used that $s\ll1$ for the last approximation on the right hand side. Using this equation, we can calculate the ratio of seeping times in the two environments:

$$\frac{T_{s,batch}}{T_{s,chemostat}}\approx\frac{\frac{2}{s}\ln N_{0}}{\frac{2}{s}\ln N_{c}}=\frac{\ln N_{0}}{\ln N_{c}}=\frac{2}{3},$$

where we have used that $N_{0}={10}^{6}$ and $N_{c}={10}^{9}$. Taken together, we see that for the total fixation time in the batch culture we obtain:

$$T_{fix,batch}=T_{m,batch}+T_{s,batch}\approx21{\cdot T}_{m,chemostat}+\frac{2}{3}T_{s,chemostat}. (5)$$

Thus, the difference in fixation times of beneficial mutations in the batch culture and chemostat is dominated by the larger number of mutations that need to be sampled in a batch culture before a mutation emerges that fixates in the population.

To roughly estimate the expected number of generations it would take for the for a beneficial mutation to reach fixation in the chemostat, we consider a mutation that has a selection coefficient $s=0.1$. For comparison, this mutation would decrease the doubling time of a *bem3Δnrp1Δ* mutant from $T_{d}=110$ to $T_{d}=100$ minutes. The time for a *de novo* generated mutation with this selection coefficient to arise in the population can be estimated by:

$$T_{m,chemostat}=\frac{1}{N_{c}\cdot U_{B}\cdot\rho\left( s \right)\cdot P\left( chemostat \right)}\approx\frac{1}{N_{c}\cdot U_{B}\cdot\rho\left( s \right)\cdot2s}.$$

Here, $U_{B}$ is the rate at which beneficial mutations occur per cell division and $\rho(s)$ is the probability that a beneficial mutation has a selection coefficient of $s$ or higher. To determine $\rho(s)$, we assume that the distribution of fitness effects follows an exponential distribution (Orr, 2003; Kassen and Bataillon, 2006; Good et al., 2012; Barlukova and Rouzine, 2021) with a mean of $\sigma=0.01$:

$$\rho\left( s \right)=\int_{s}^{\infty} \frac{1}{\sigma}e^{-s/\sigma}.$$

Taking the beneficial mutation rate to be of the order $U_{B}\sim{10}^{-5}$ per cell per generation, we obtain the result that the expected number of generations it will take for a beneficial mutation to occur that escapes drift is

$$T_{m,chemostat}\sim11 \mathrm{generations}.$$

The time it takes for this mutation to sweep the population can be calculated using equation 4, which for $s=0.1$ gives:

$$T_{s,chemostat},chemostat\sim207 generations.$$

These results show that while we expect that beneficial mutations arise after only few generations in the chemostat, these mutations sweep through the population at a relatively slow rate. Converting the values of $T_{m}$ and $T_{s}$ of the chemostat to the expected values for the batch culture using equation 5 results in:

$$T_{m,batch}\sim231 \mathrm{generations}$$

and

$$T_{s,batch}\sim138 generations.$$

Thus, the situation is reversed in the batch culture: beneficial mutations emerge only sparingly due to the loss of genetic variation caused by the population bottlenecks, but once they emerge these mutations sweep through the population faster than in a continuous culture. This result is in agreement with our finding that *bem3Δnrp1Δ* populations evolved through serial dilutions show no significant difference in their phenotype compared to their ancestor after 70 generations. Comparing the total fixation times to the true number of generations that we evolved the *bem3Δnrp1Δ* populations in the two conditions indicates that beneficial mutations in the batch culture (evolved for 300 generations, expected $T_{fix}$= 369 generations) are expected to be closer to fixation than beneficial mutations in the chemostat (evolved for 70 generations, expected $T_{fix}$= 218 generations). An important remark is that in our calculation of the fixation times, we have assumed that beneficial mutations are rare enough that they only emerge in succession and never simultaneously, thereby ignoring clonal interference (Gerrish and Lenski, 1998; Desai and Fisher, 2007; Desai et al., 2007). Clonal interference is likely to play a larger role in continuous culture than in batch cultures due to the larger amount of genetic variation and may further reduce the speed of evolution.

**Estimation of population sizes**

The approximate population sizes were determined from the optical density (OD600) measurements taken of the growing populations in the two environments. These measurements show that the OD600 is $\sim3$ for populations growing in the chemostat and $\sim10$ for populations growing in the batch culture just before serial passaging. Using the approximation that a population with an OD600 of 1 contains of the order ${10}^{7}$cells/ml (Day et al., 2004) and the total culture volumes of 20 ml for the chemostat and 10 ml for the batch culture, we find that both the chemostat and batch culture populations contain of the order of ${10}^{9}$ individuals.

**References**

Antal, T., and Scheuring, I. (2006). Fixation of Strategies for an Evolutionary Game in Finite Populations. *Bulletin of Mathematical Biology* 68(8)**,** 1923-1944. doi: 10.1007/s11538-006-9061-4.

Barlukova, A., and Rouzine, I.M. (2021). The evolutionary origin of the universal distribution of mutation fitness effect. *PLOS Computational Biology* 17(3)**,** e1008822. doi: 10.1371/journal.pcbi.1008822.

Campos, P.R.A., and Wahl, L.M. (2009). THE EFFECTS OF POPULATION BOTTLENECKS ON CLONAL INTERFERENCE, AND THE ADAPTATION EFFECTIVE POPULATION SIZE. *Evolution* 63(4)**,** 950-958. doi: 10.1111/j.1558-5646.2008.00595.x.

Day, A., Schneider, C., and Schneider, B.L. (2004). "Yeast Cell Synchronization," in *Cell Cycle Checkpoint Control Protocols,* ed. H.B. Lieberman. (Totowa, NJ: Humana Press), 55-76.

Desai, M.M., and Fisher, D.S. (2007). Beneficial Mutation–Selection Balance and the Effect of Linkage on Positive Selection. *Genetics* 176(3)**,** 1759-1798. doi: 10.1534/genetics.106.067678.

Desai, M.M., Fisher, D.S., and Murray, A.W. (2007). The Speed of Evolution and Maintenance of Variation in Asexual Populations. *Current Biology* 17(5)**,** 385-394. doi: <https://doi.org/10.1016/j.cub.2007.01.072>.

Gerrish, P.J., and Lenski, R.E. (1998). The fate of competing beneficial mutations in an asexual population. *Genetica* 102(0)**,** 127-144. doi: 10.1023/A:1017067816551.

Good, B.H., Rouzine, I.M., Balick, D.J., Hallatschek, O., and Desai, M.M. (2012). Distribution of fixed beneficial mutations and the rate of adaptation in asexual populations. *Proceedings of the National Academy of Sciences* 109(13)**,** 4950-4955. doi: 10.1073/pnas.1119910109.

Haldane, J.B.S. (1927). A Mathematical Theory of Natural and Artificial Selection, Part V: Selection and Mutation. *Mathematical Proceedings of the Cambridge Philosophical Society* 23(7)**,** 838-844. doi: 10.1017/S0305004100015644.

Kassen, R., and Bataillon, T. (2006). Distribution of fitness effects among beneficial mutations before selection in experimental populations of bacteria. *Nature Genetics* 38(4)**,** 484-488. doi: 10.1038/ng1751.

M Altrock, P., and Traulsen, A. (2009). Fixation times in evolutionary games under weak selection. *New Journal of Physics* 11(1)**,** 013012. doi: 10.1088/1367-2630/11/1/013012.

Orr, H.A. (2003). The Distribution of Fitness Effects Among Beneficial Mutations. *Genetics* 163(4)**,** 1519-1526. doi: 10.1093/genetics/163.4.1519.

Tkadlec, J., Pavlogiannis, A., Chatterjee, K., and Nowak, M.A. (2019). Population structure determines the tradeoff between fixation probability and fixation time. *Communications Biology* 2(1)**,** 138. doi: 10.1038/s42003-019-0373-y.

Wahl, L.M., Gerrish, P.J., and Saika-Voivod, I. (2002). Evaluating the impact of population bottlenecks in experimental evolution. *Genetics* 162(2)**,** 961-971.

## **Supplementary figures**


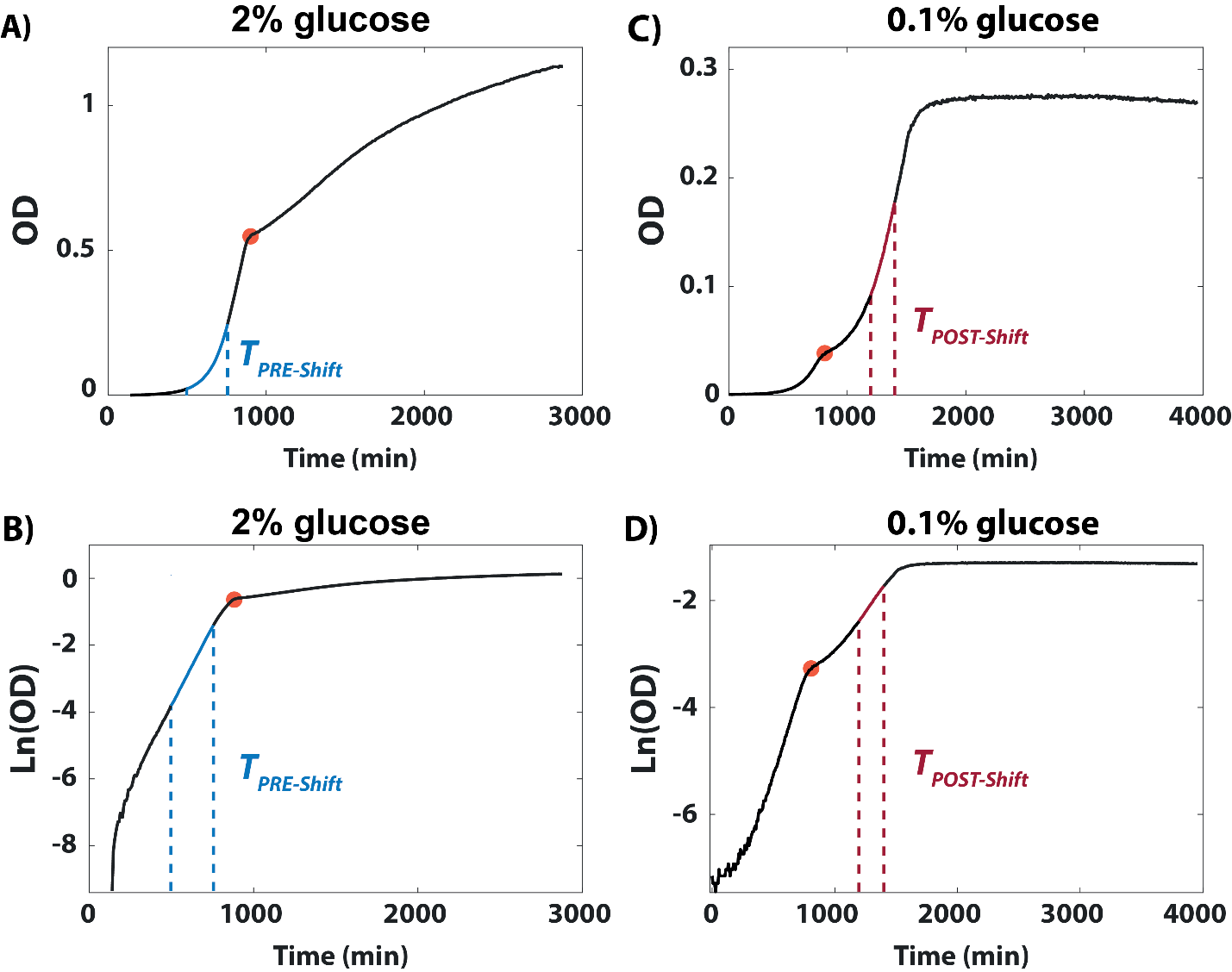


Figure S1. Low glucose media induces earlier diauxic shift, allowing better quantification of growth after the diauxic shift. (A-B) When grown on high glucose (2%) media, most of the biomass is produced by fermentation. (C-D) Lowering the glucose concentration to 0.1% induces an earlier diauxic shift and improves visualization and quantification of growth after the shift. Dashed lines indicate the period of exponential growth for growth in YP+2% glucose (blue) and for growth in YP+0.1% glucose (red). Although the diauxic shift phase is clearly visible in both cases as a temporary cessation of growth (indicated by the red dots), the high OD at which the diauxic shift occurs in 2% glucose media makes it unsuitable to quantify growth beyond this point due to the possible non-linear relationship between cell density and absorbance at high OD values. Conversely, in 0.1% glucose media the diauxic shift occurs at a relatively low density, decreasing the amount of signal relative to the noise during pre-diauxic growth. Therefore, population doubling times before passage through the diauxic shift(*T_PRE-Shift_*) were determined from growth data in 2% glucose media, while population doubling times after passage through the diauxic shift (*T_POST-Shift_*) were obtained from growth data 0.1% glucose media.


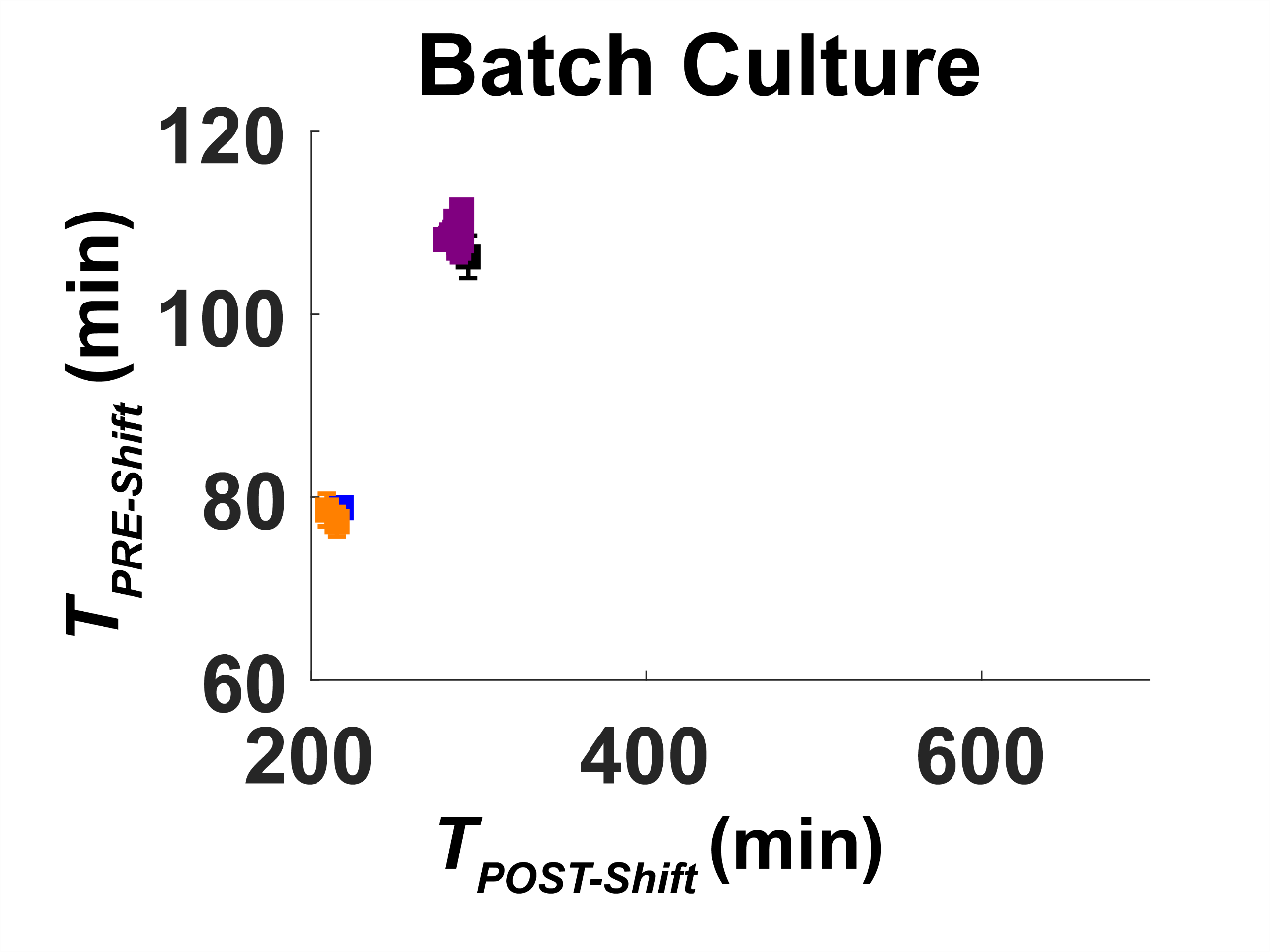


Figure S2. Scatter plot of *T_PRE-Shift_* vs *T_POST-Shift_* after 70 generations in a batch culture. After 70 generations of evolution in a batch culture the evolved cell lines were still phenotypically highly similar to their ancestral strains. This was the case for both the *bem3∆nrp1∆* mutants and the WT strains.


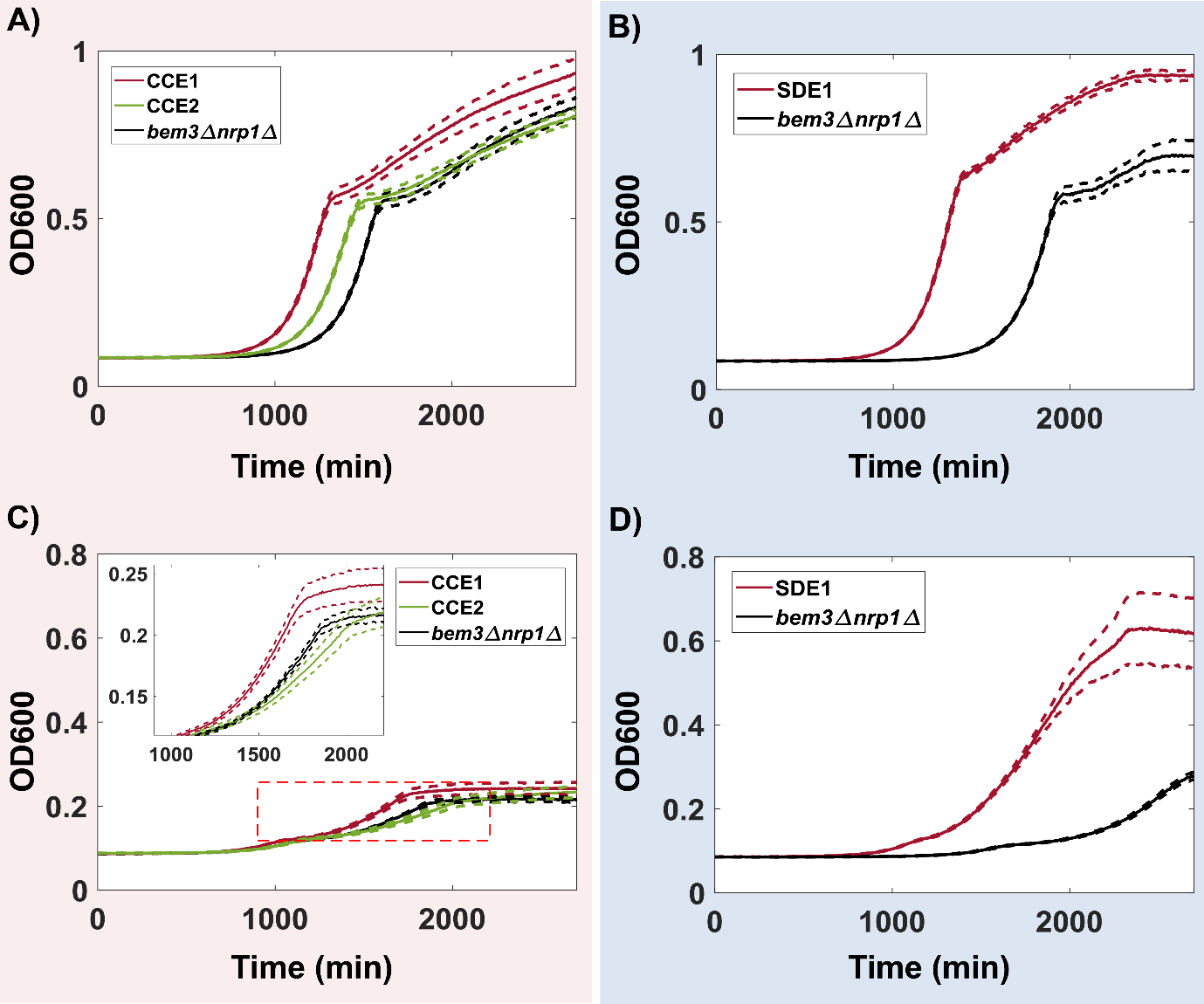


Figure S3 . Growth curves of selected evolved lines CCE1, CCE2 and SDE1. (A,C) Bulk measurements in (A) 2% dextrose and (C) 0.1% dextrose media of selected evolved mutant lines CCE1 and CCE2 from the continuous culture experiment together with the mutant ancestor strain. (B,D) Bulk measurements in (B) 2% dextrose and (D) 0.1% dextrose media selected evolved mutant line SDE1 from the batch culture experiment. Dashed lines display the SEM.
