# Supplementary figures and images for: Pleiotropy drives evolutionary repair of the responsiveness of polarized cell growth to environmental cues
Source: Front Microbiol. 2023 Jul 14;14:1076570. doi: 10.3389/fmicb.2023.1076570 (PMC10382278; doi:10.3389/fmicb.2023.1076570)

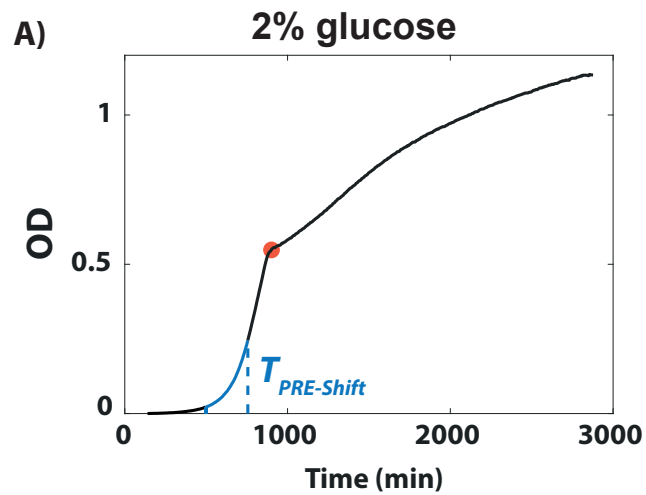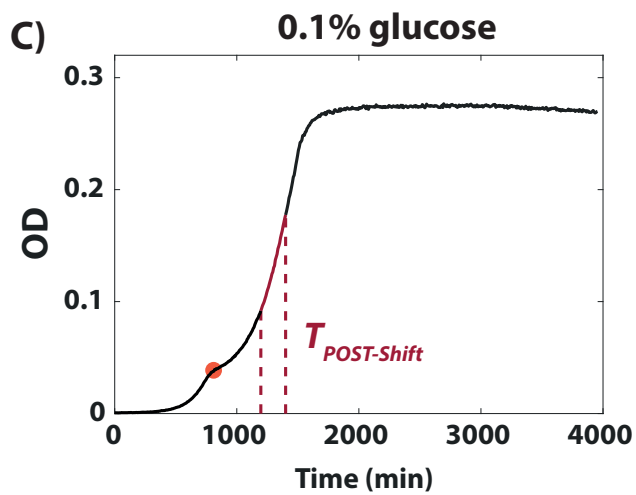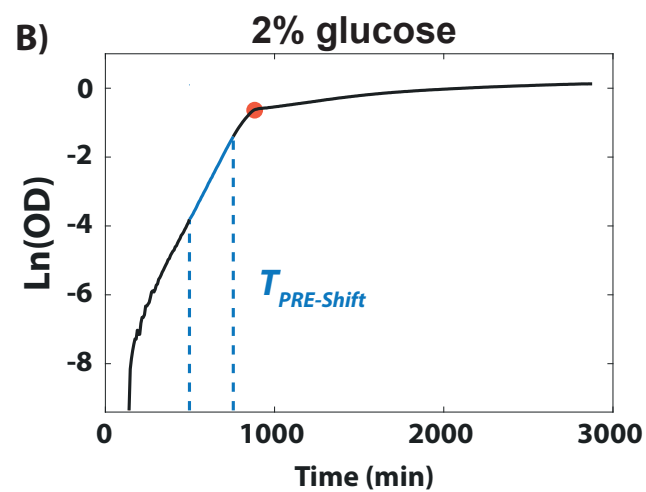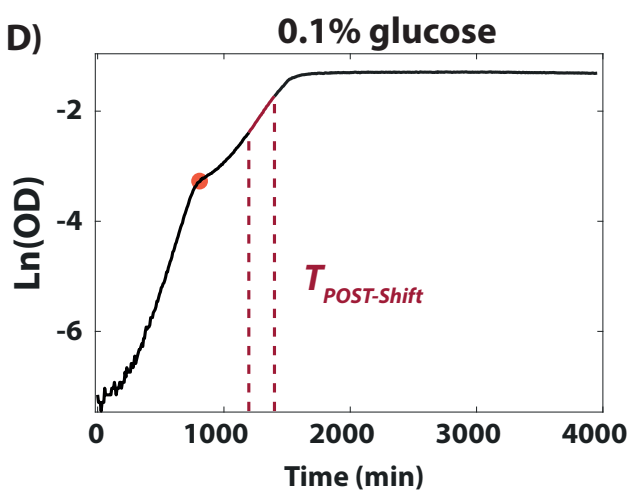

Supplement: Supplementary file 4 [file Image_1.pdf]

## Serial Dilutions

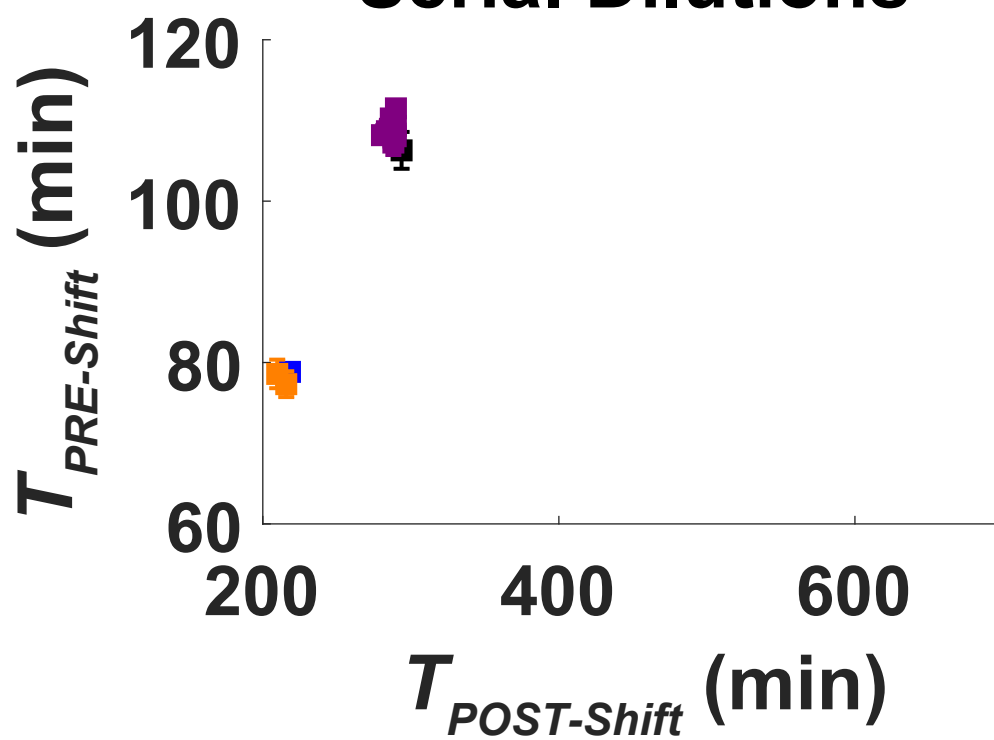

Supplement: Supplementary file 5 [file Image_2.pdf]

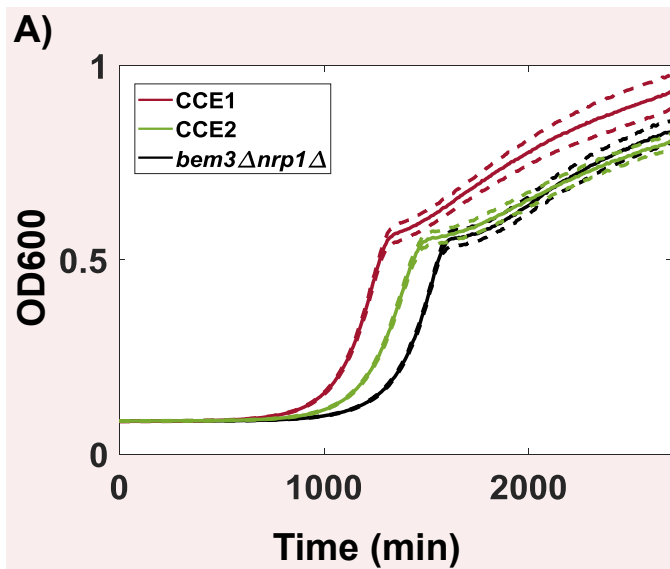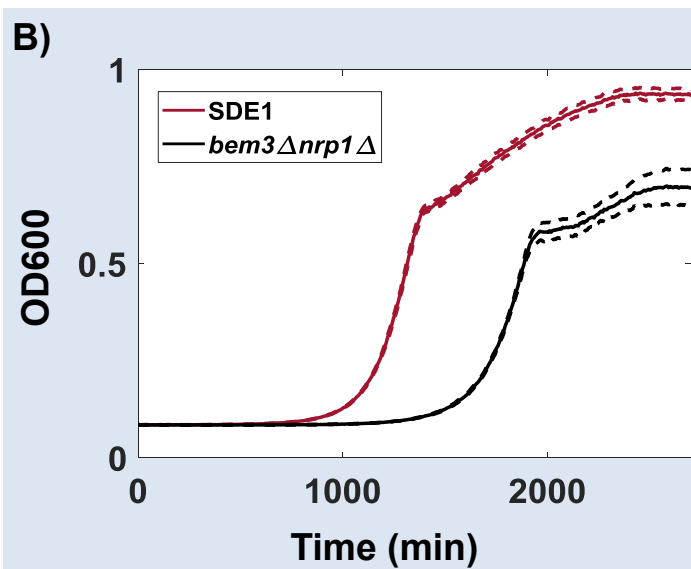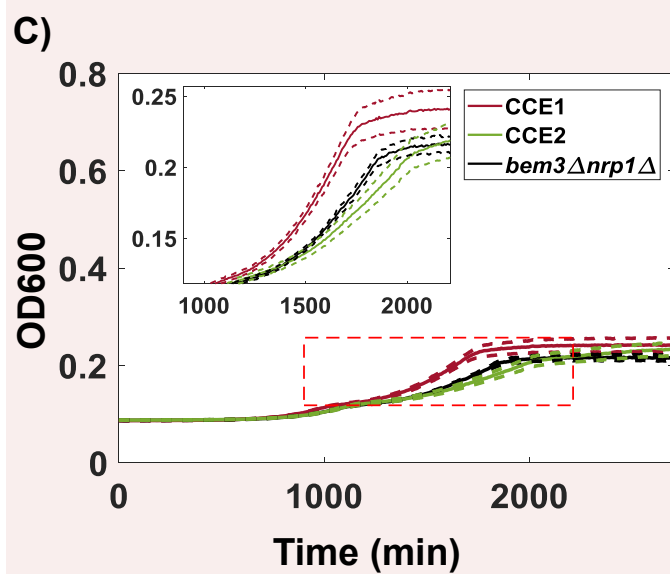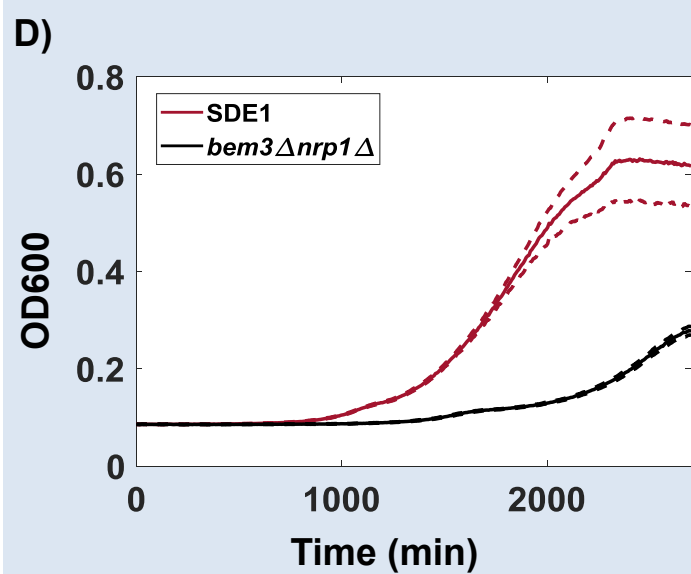

Supplement: Supplementary file 6 [file Image_3.pdf]
